# Case report: Delayed autologous tooth transplantation based on objective bone healing of the extraction socket (4-year follow-up)

**DOI:** 10.3389/fdmed.2023.1061362

**Published:** 2023-06-15

**Authors:** Yusuke Takahashi, Shotaro Abe, Motoki Okamoto, Tomomi Tsujimoto, Shumei Murakami, Mikako Hayashi

**Affiliations:** ^1^ Department of Restorative Dentistry and Endodontology, Osaka University Graduate School of Dentistry, Osaka, Japan; ^2^ Department of Oral Science and Translational Research, College of Dental Medicine, Nova Southeastern University, Fort Lauderdale, FL, United States; ^3^ Department of Oral and Maxillofacial Radiology, Osaka University Graduate School of Dentistry, Osaka, Japan

**Keywords:** autologous tooth transplantation, delayed transplantation, multi-detector row computed tomography (MDCT), computed tomography (CT) value, bone healing

## Abstract

Autologous tooth transplantation is one of the best methods to replace a missing tooth when there is a suitable donor tooth. Tooth transplantation is mainly performed immediately after extraction because this is completed in a single surgery and the donor tooth is transferred to fresh recipient site facilitated by the remaining periodontal ligament. However, when transplantation is planned for a severe recipient site with a large bone defect surrounding the affected tooth, delayed transplantation is performed because of the mismatched size of the donor tooth. When bone formation at the recipient site is gradually observed during wound healing, transplantation can be performed. However, the estimated time for delayed transplantation has not been clearly determined because of the varied wound healing at the recipient site. This case report demonstrates successful tooth transplantation 4 months after extraction by monitoring bone healing of the recipient site by computed tomography (CT). A male patient complained about occlusal pain in his mandibular molar. He had received the latest restoration after root canal treatment 10 years previously. Seven years later, he experienced slight spontaneous pain and consulted a private dental clinic. Radiographic examination revealed vertical root fracture and the dentist recommended tooth extraction, but he did not receive this suggestion. Several years later, he visited our hospital, and the bone resorption became much larger, and the surrounding bone was completely lost. Thus, it was decided that autologous tooth transplantation should occur several months later because of the poor fit of the donor tooth using wisdom tooth. Sequential CT value was monitored during bone formation at the recipient site by multi-detector computed tomography. Four months later, the CT value of the recipient site had gradually increased and tooth transplantation was performed. Fit of the donor tooth to the recipient site was still poor at the surgery, but it became better and tooth mobility decreased gradually. After performing root canal treatment, final full covered restoration was equipped. Review at 4 years after transplantation revealed the tooth showed no symptoms with no apical radiolucency. This case report suggests that delayed tooth transplantation can be performed after monitoring bone formation at the recipient site by x-ray or CT images.

## Introduction

The concept of minimal intervention dentistry (1) has become popular among dentists recently, and many dentists have used least invasive treatment methods mainly in restorative dentistry (2). Prosthodontic treatment for missing teeth has several options including a removable/fixed denture or dental implant. These treatments have certain survival rates for long periods (3–5). Autologous tooth transplantation is another option to fill a missing tooth and is considered to be a minimal intervention method compared with a dental implant when the patient has teeth for transplantation (6). Autotransplantation is regarded as a better option than an implant because of the periodontal ligament of the donor tooth, which is absent in an implant (7). Thus, transplanted tooth doesn't need a special maintenance like implants because it can be dealt like natural tooth. Transplantation has relatively low risk concerning invading the mandibular canal when the canal is close to the recipient site compared with dental implant which needs specific drilling, this also can be a kind of minimal intervention. The success rate of implants is approximately 95% after 5 years (8), while autotransplantation results in approximately 90% success (9, 10).

In many cases, immediate autotransplantation is planned to be performed because this surgery can be completed in a single operation and the fresh periodontal ligament from both the donor tooth and recipient socket can be used. However, a large bone defect surrounding a recipient site may cause a failure of immediate transplantation because of the extremely poor fit of the donor tooth and recipient site. Some reports have indicated that guided bone regeneration (GBR) can be performed in such cases (11, 12), but there has been limited clinical evidence for GBR by natural/artificial bone in autologous transplantation. Thus, delayed transplantation can be performed in such cases. Ferreira reported there was no significant difference between the immediate transplantation and 7-day delayed transplantation in terms of wound healing observed in dog sound tooth model (13). This period should be determined by the wound healing stage of the recipient site, and transplantation may be successful when bone formation increases. If the recipient site is under severe inflammatory condition or poor fit of donor tooth, this period can be longer.

This case report presents a successful delayed autologous tooth transplantation by monitoring bone formation quantitively at the recipient site using computed tomography (CT), depending on the shift of the CT value under informed consent of the patient.

## Case description

A 53-year-old male patient with occlusal pain in the mandibular right second molar was referred to the Department of Restorative Dentistry and Endodontics (Osaka University Dental Hospital, Osaka, Japan) by a dentist from a private dental clinic. He had received a cast metal restoration with a metal post core after root canal treatment 10 years previously. This tooth was asymptomatic for several years. He then experienced slight spontaneous pain and consulted a private clinic. He received a regular radiographic examination that revealed a vertical root fracture at this tooth. The dentist recommended extraction because of its poor prognosis, but the patient declined because the pain was mild at that time. Three years later, he visited the same clinic with occlusal pain. The radiographic examination indicated much larger bone resorption around the tooth, and the dentist diagnosed that this tooth would be lost and recommended an implant after extraction with GBR application. The dentist removed the metal restoration, confirmed the root fracture, and temporarily sealed the fracture with a resin composite. The patient had wisdom teeth and asked the dentist whether it was possible to perform a tooth transplantation, but the dentist did not have much experience with tooth transplantation. Then, he visited our hospital with a referral letter. The patient was a non-smoker and took no specific medication. He did not have any specific medical or family history.

**Figure 1:**
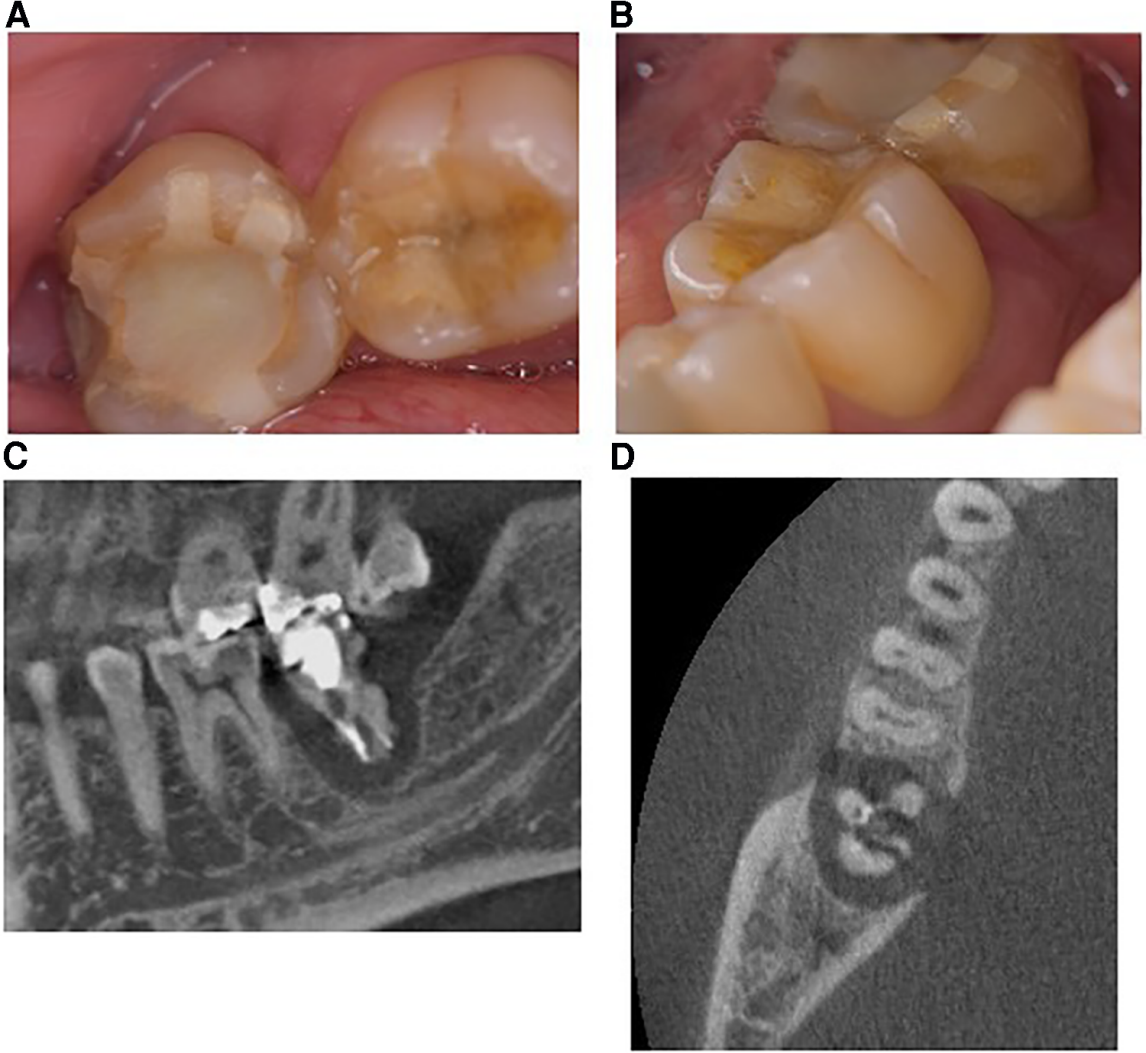
Images obtained at the first visit to the university clinic. (**A,B**) Intraoral images. Right mandibular second molars with a temporary resin composite filling showed slight gingival swelling between the first molar. The probe depth on the distal side was 12 mm and the tooth showed slight mobility. (**C**) Selected sagittal plane of multidetector computed tomography (MDCT). The root was fractured and the surrounding alveolar bone was completely defected. (**D**) Axial plane of MDCT. The root also indicated a fractured condition and the surrounding bone was widely resorbed.

Extraoral examination in our hospital revealed no facial swelling or asymmetry, whereas an intraoral examination revealed slight buccal gingival swelling without the sinus tract in the region of the right mandibular molar (Figures 1A,B). The periodontal probe depth was 12 mm at the distal side with M1 mobility. The tooth demonstrated slight pain to percussion and palpation.

**Figure 2:**
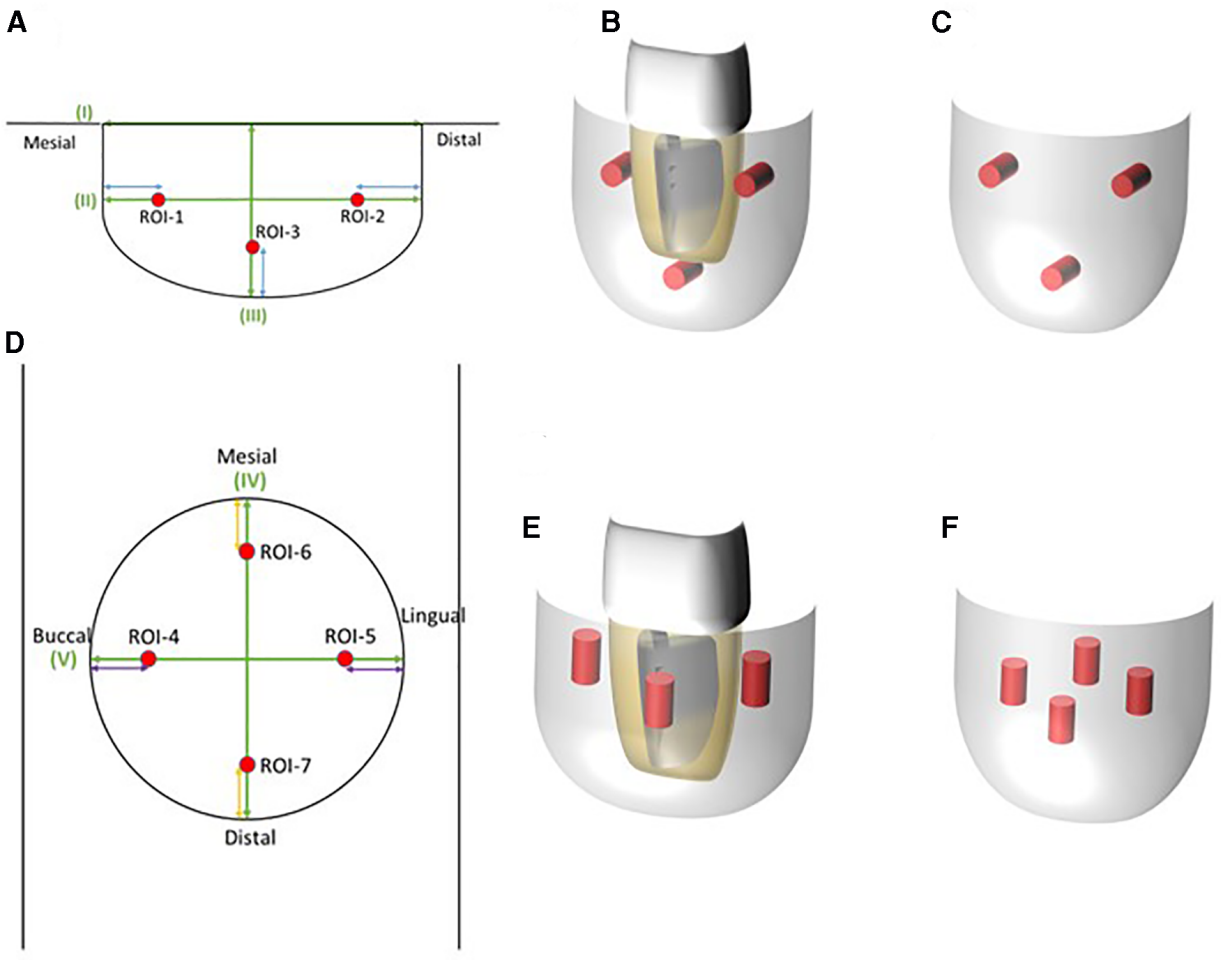
Regions of interest (ROI) set to evaluate bone healing by measuring the CT value obtained from MDCT. (**A**) Image of an ROI in the sagittal plane of MDCT. Three points were set to measure the CT value. (**B**) ROI 3D images of the sagittal plane where the donor tooth could be positioned. Red bars were considered to be closely placed on the root surface of the donor tooth. (**C**) ROI 3D image without the donor tooth. (**D**) ROI image of the axial plane in MDCT. Four points were placed for CT value evaluation. (**E**) ROI 3D image of the axial plane where the donor tooth might be positioned. Bars were positioned around the root surface of the donor tooth. (**F**) ROI 3D image without the donor tooth. CT value at each plane was the average CT value from each ROI.

**Figure 3:**
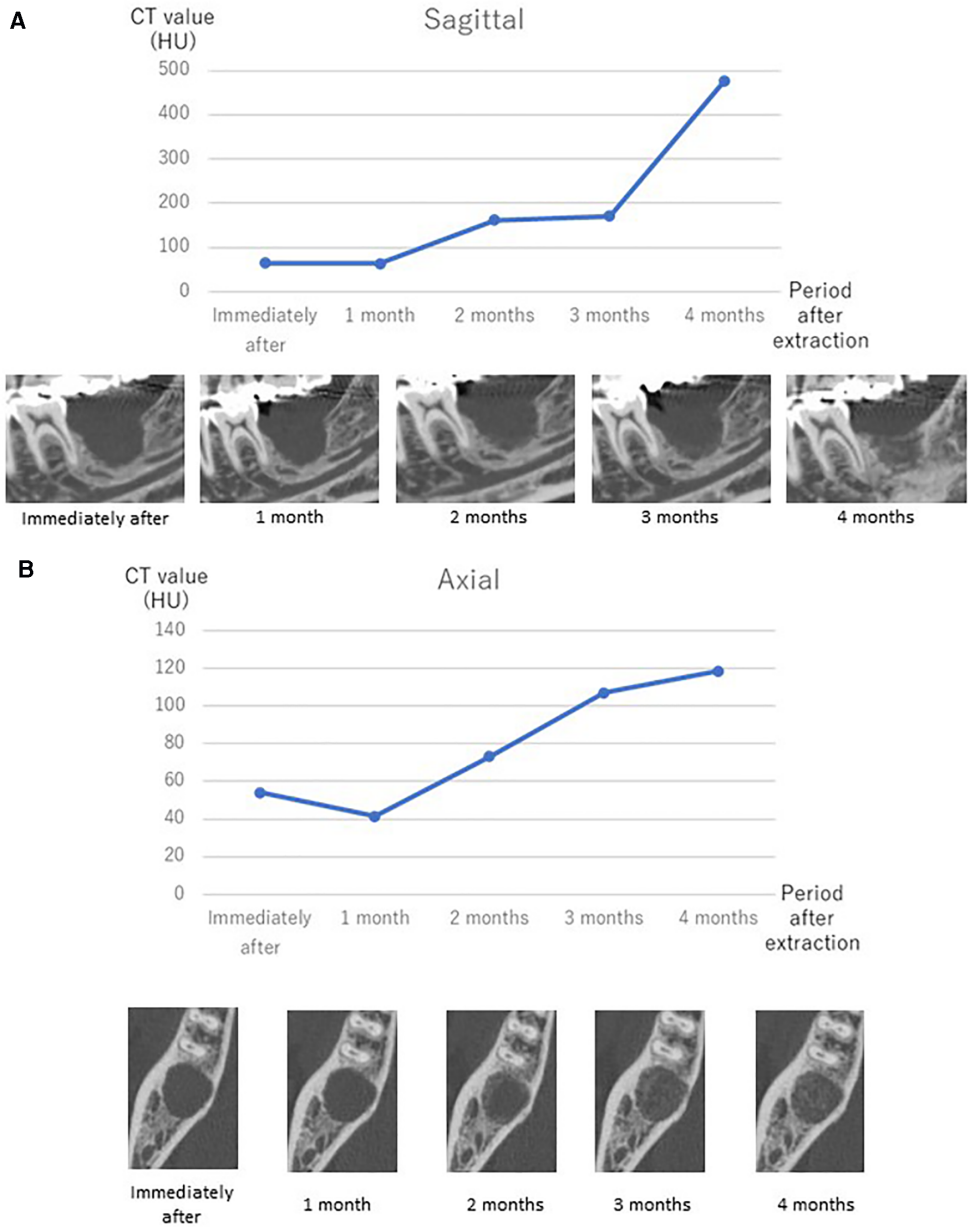
CT value shift after extraction of the affected tooth measured by MDCT and representative MDCT images at each time point in the sagittal plane (**A**) and axial plane (**B**). Transplantation was performed after confirmation of the increasing tendency of the CT value at 4 months after extraction of the affected tooth.

**Figure 4:**
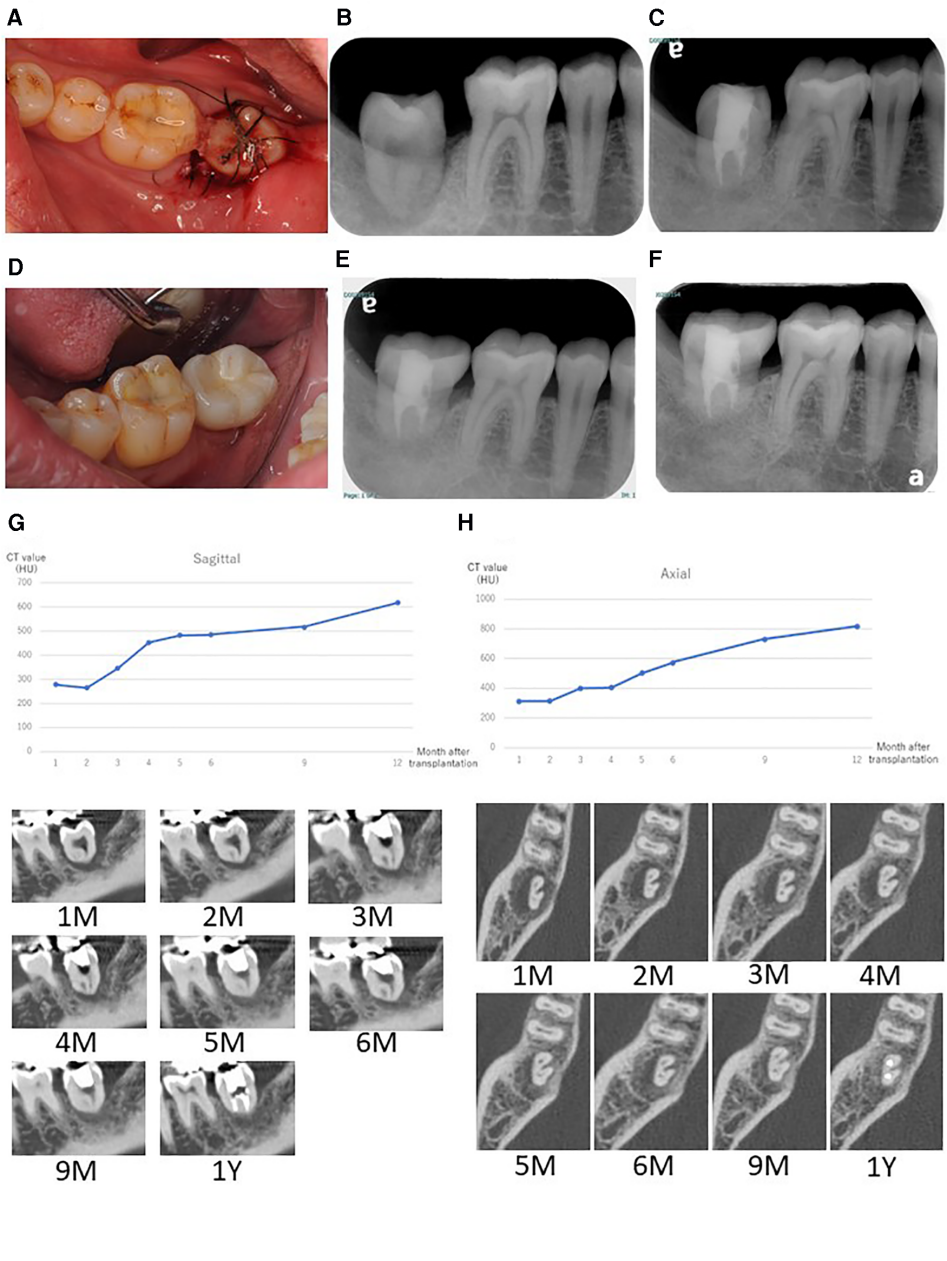
(**A**) Intraoral image after transplantation. Fixation was performed using a silk suture and adhesive resin cement. (**B**) Radiographic image at 2 weeks after transplantation. Fit of the transplanted tooth was still low at this stage. (**C**) Radiographic image at 1 year after the transplantation. The tooth was completely surrounded by alveolar bone and horizontal bone resorption was not obvious. (**D**) Intraoral image at 1 year after transplantation with the final restoration. Radiographic image at 2 years (**E**) and 4 years (**F**) after transplantation. CT value shift and representative images after tooth transplantation up to 12 months in the sagittal plane (**G**) and axial plane (**H**). CT-value in each graph was calculated as the average CT-value from each ROI.

Multi-detector computed tomography (MDCT) images were acquired (Light Speed VCT; GE Healthcare, Milwaukee, WI, USA) at 120 kVp and 210–330 mA. The field of view was 25 cm × 25 cm, the matrix size was 512 × 512, and the slice thickness was 0.625 mm with no interslice spacing. Images were displayed with the bone window [WW/WL: 4,000/800 Hounsfield units (HU)]. MDCT revealed complete loss of the supporting bone around the affected tooth which was less than 100 HU (Figures 1C,D) and apparent root fracture. From the results of the MDCT images, it was decided that this tooth could no longer be conserved. After extraction of the tooth, we suggested several prosthodontic options as follows: (i) no treatment after extraction, (ii) implant with GBR, (iii) fixed/removable dental prostheses, or (iv) delayed autologous tooth transplantation. The patient chose tooth transplantation. Because the patient had wisdom teeth, we decided to perform a tooth transplantation. An implant could be used combined with GBR, but the patient did not choose this option. He rejected a fixed cantilever denture because the right mandibular first molar and second premolar were almost intact. Additionally, the patient did not choose a removable denture because of its uncomfortable characteristics. Therefore, we decided to perform an autologous tooth transplantation several months later because of the poor fit of the donor tooth. Before tooth extraction, a right maxillary wisdom tooth was chosen as a donor tooth because the shape of the root was relatively straight and the patient had both upper and lower wisdom teeth on the left. For higher success, it could be suggested that evaluating the bone healing by quantitatively monitoring the sequential CT value during wound healing and bone formation at the recipient site using MDCT after extraction to determine when the tooth transplantation should be performed. The patient accepted this suggestion and agreed to attend review appointments. Cone beam computed tomography (CBCT) was not used in this case because CBCT does not generate exact CT values and it was difficult to precisely quantitate the bone healing after tooth extraction. Therefore, a delayed tooth transplantation was performed when bone healing at the recipient site was observed by MDCT. To evaluate the CT value at the recipient site, three regions of interest (ROI) in the sagittal plane (Figures 2A–C: ROI-1, -2, -3) and four ROIs in the axial plane of the MDCT images were set (Figures 2D–F: ROI-4, -5, -6, -7), where the donor tooth root was potentially located and observed them each month. CT value was measured from 5 points in each ROI site, and then the average of these 5 values was calculated as the representative CT value of each ROI. The average value of ROI-1 to -3 or ROI-4 to -7 was then calculated at each time point for the representative CT value of each plane. As a result, 4 months after the extraction, an increase in the CT value at the recipient site in both sagittal and axial planes was observed (Figures 3A,B). Then, the tooth transplantation was performed using a right maxillary wisdom tooth (14). An incision was made on the alveolar crest and then a light curettage was performed in the recipient site to ensure that not too much bony tissue was removed. The donor tooth was extracted using forceps and transplanted into the recipient site with 90° rotation within 10 min after the extraction. The fit of the donor tooth to the recipient site was still relatively poor at the surgery. The tooth was fixed using a silk suture and adhesive resin cement (SuperBond C&B, Sun Medical, Shiga, Japan) (Figures 4A,B) to the proximal tooth. Tooth mobility decreased gradually until 1.5 months. A root canal treatment of the transplanted tooth was performed 2 months after the surgery (Figure 4C). Then, a final full coverage restoration was equipped (Figure 4D). After the transplantation, MDCT images were obtained every month until 6 months, and at 9, and 12 months (Figures 4G,H). The CT value around the transplanted tooth had gradually increased until 1 year after the surgery. Review at 2 (Figure 4E) and 4 years (Figure 4F) after transplantation revealed that the tooth showed no symptoms or apical radiolucency. Timeline of the current case is shown in Table 1.

**Table 1:** Timeline of symptoms and treatment of autotransplantation.

	Clinical symptoms	Clinical treatment
Three years previously (private dental clinic)	Slight spontaneous pain Detection of root fracture	Observation
One month previously (private dental clinic)	Occlusal pain Large bone defect	Removal of metal crown Confirmation of fracture
1st visit (at dental hospital)	Sligh gingival swelling Periodontal probing depth of 12 mm at distal M1 mobility Slight pain to percussion and palpitation	Diagnosis Treatment plan Consultation
2nd visit	Large bone defect in MDCT images	MDCT before treatment Determination of treatment plan (Delayed autotransplantation)
3rd visit		Extraction of right mandibular second molar MDCT immediately after extraction
	Wound healing	MDCT (observing bone formation at recipient site)
4 months after the extraction	In the middle of wound healing (CT value: around 500HU)	Autologous tooth transplantation from right maxillary wisdom tooth
2 months after the surgery	Decreased tooth mobility (M0-1)	Start root canal treatment
3, 4, 5, 6, 9, and 12 months after the surgery	No tooth mobility	MDCT (observing bone formation) Final restoration
2 and 4 years after the surgery	No symptoms	Follow-up

## Discussion

The survival rate of prosthodontic treatments for missing teeth has been widely reported, but most studies have been independent assessments of each treatment option (3, 9, 15–17). Implants have a high success rate at >95% for 5 years (3) and 87.8% for a minimum of 20 years (15). Fixed dental prostheses have a 94.4% success rate for 5 years depending on the materials used (16). Removable prostheses have 50%–67% success rates for 5 years, but this report evaluated broad cases, such as different dentition or occlusal style (17). In terms of autologous transplantation, the survival rate is 75.3%–91% for 6 years or more (9). There have been some reports that directly compared these treatment options. Comparisons of implants and fixed dental prostheses have shown that the survival rate per year of the implants is 99% and that of fixed dental prostheses is 97.9% (4). Another report compared survival rates among implants (94.7%), fixed dental prostheses (77.4%), and removable dentures (33.3%) for 6 years, but such comparisons in a clinical situation might describe a tendency (5). A review article indicated that multiple risk factors may affect the prognosis of treatments, such as host-derived, bacterial infection-derived, load-associated, or technical factors, and each parameter may be closely associated with each other (18). These survival rates cannot be simply compared, but tooth autotransplantation is an option for missing teeth from the view of minimal intervention, beneficial use of the preserved periodontal ligament, and cost-effectiveness. Additionally, even if the transplantation eventually fails, the surrounding tissue may be a candidate for subsequent implant treatment (19).

In the current case, autologous tooth transplantation was performed using a maxillary wisdom tooth into the recipient site of the mandibular right second molar with a large bone defect. This bone defect was very large (Figures 1C,D), and an immediate tooth transplantation was considered to be difficult to perform. In addition, the distance from the bottom of the recipient site to the mandibular canal was quite small, dental implant could be a high-risk procedure in this case (Figures 1C, 3A). However, there are no defined criteria or guidelines to increase the success rate of autotransplantation in such a case. Thus, a delayed transplantation was performed, but there was limited information concerning when a delayed transplantation should be performed. Tsukiboshi indicated that a delayed tooth transplantation should be performed within 2–6 weeks after tooth extraction from the recipient site because extensive bone resorption may occur 6 weeks after the extraction (20). This previous study also indicated that the timing should be determined by whether the gingival flap covers the surrounding area of the donor tooth after extraction from the recipient site. If the gingival soft tissue does not sufficiently cover the area to close the surgical site, an infection may occur. In the current case, a delayed transplantation was performed 4 months after extraction, and horizontal bone resorption was observed to some extent (Figure 4B). Additionally, gingival tissue at the recipient site had completely healed in this case. Another report compared the survival rate of autotransplantation in fresh and surgically created sockets, and there was no significant difference. It was also indicated that autotransplantation combined with GBR did not affect the success rate of either immediate or delayed transplantation (12, 13). However, these report assessed surgically created sockets that were sound bone tissue and quite different from the current case with a large bone defect at the recipient site. In this case, preoperative bone resorption was so wide and lost even lingual supporting alveolar bone, thus GBR might be technically difficult and also high risk of post-operative infection. If GBR was applied in this case, the horizontal alveolar bone level could be higher, but it was still a challenging procedure. It is necessary to accumulate more clinical evidence in this field. Other techniques for achieving better outcomes, root surface treatment of the donor tooth could be considered. Laser irradiation or some enzymatic treatment was reported to improve the characteristics of the root surface (21, 22). Effective surface treatment of the implant fixture was also reported (23). Accumulation of basic and clinical evidence can facilitate higher success rate of autotransplantation. We focused on the CT value recovery/increase in the recipient site where the root surface of the donor tooth would be positioned after transplantation (Figure 2). The CT value of the recipient site was sequentially measured immediately after tooth extraction each month for 4 months with informed consent from the patient. Then, the CT value had gradually continued to rise over time to approximately 500 HU in the sagittal plane view. This value indicated approximately half of a representative bone cortex (24) and it was considered that this stage could be under the wound healing in addition to vascular reformation and better nutritional condition and good timing for transplantation (13). During the surgery, a light curettage was performed to prepare the recipient. Some bony tissue such as hard tissue fragments were observed and relatively soft compared with the cortical bone. This suggested that the recipient site was undergoing bone formation and matched the CT value. As a result, the autotransplantation surgery was successfully completed. It can be difficult to take MDCT images frequently under the usual dental clinical condition, but CBCT or regular x-ray images can be obtained for an approximate reference of bone healing to determine when autotransplantation should be performed.

Proper timing for a root canal treatment after transplantation has also not been determined. Fouad et al. reported that root canal treatment can be started 2 weeks after replantation for an avulsed tooth as a guideline to manage traumatic dental injuries (25). This information can be used as a background to determine the timing of when to start a root canal treatment after transplantation. In the current case, root canal treatment started 2 months after autotransplantation because adaptation of the transplanted tooth was relatively poorer than that of the replanted tooth. To avoid physical damage to the periodontal tissue of the transplanted tooth, 2 months might have been the proper timing in this case because of the large bone defect. For younger patient with immature root apex of the donor tooth, pulp tissue can be survived or regenerated (26–28). However, age of the patient in the current case was over 50s and necrotic pulp tissue could be a risk of inflammatory root resorption (29). Thus, root canal treatment was performed to avoid such phenomenon. In the future, contemporary pulp regenerative treatment may be performed at transplanted tooth (30).

The CT value at the ROI around the transplanted tooth was sequentially observed until 1 year after the surgery. The value had gradually increased from both sagittal and axial views of MDCT (Figures 4F,G), and this tooth showed no clinical complications after 2 and 4 years (Figures 4E,F). This indicated that the procedure was successful in this case. It is necessary for longer follow-up for accumulation of a clinical evidence from this case.

This case report could show successful delayed autotransplantation performed 4 months after the extraction which was quite different from the previous report (18). This case can grant several months for a delayed transplantation depending upon the size of bone defect, wound healing capacity of the host. When performing delayed transplantation for a patient with systemic disease, it may be difficult to predict the bone resorption and the sequential monitor of bone healing using MDCT like this case can be considered.

The limitation of this study was that the high frequency of taking MDCT images. In this case report, the patient agreed to be taken MDCT, and the frequent evaluation might enable more objective and precise analysis and to lead the successful delayed autotransplantation. However, this report is one of references and it may not be necessary to take MDCT with such frequency, and clinicians can use CBCT or plain x-ray photo instead of MDCT. It may be easy to realize the sequential difference of bone formation of the recipient site by visual inspection (Figure 3). Furthermore, the long-term perspective of this case is still unclear because such delayed transplantation has not been reported, but this can be a treatment option for both dentists and patients.

## Conclusion

This case study demonstrated successful delayed autologous transplantation into a large bone defect with sequential observation of the CT value at the recipient site. Delayed transplantation can be performed by observing bone healing by visual inspection in sequential CBCT or x-ray images.

## Data Availability

The original contributions presented in the study are included in the article, further inquiries can be directed to the corresponding author.
